# Dynamic postural control correlates with activities of daily living and quality of life in patients with knee osteoarthritis

**DOI:** 10.1186/s12891-021-04164-1

**Published:** 2021-03-18

**Authors:** Kento Sabashi, Tomoya Ishida, Hisashi Matsumoto, Kentaro Mikami, Takeshi Chiba, Masanori Yamanaka, Yoshimitsu Aoki, Harukazu Tohyama

**Affiliations:** 1 Faculty of Health Sciences, Hokkaido University, Kita 12, Nishi 5, Kita-ku, 060-0812 Sapporo, Japan; 2 Department of Rehabilitation, Hokkaido University Hospital, Kita 14, Nishi 5, Kita-ku, 060-8648 Sapporo, Japan; 3 Department of Rehabilitation, Hokushin Orthopaedic Hospital, 1-5, Kita 8, Higashi 4, Higashi- ku, 060-0908 Sapporo, Japan; 4 Faculty of Health Science, Hokkaido Chitose College of Rehabilitation, Satomi 2-10, 066-0055 Chitose, Japan; 5 Department of Orthopaedic Surgery, Hokushin Orthopaedic Hospital, 1-5, Kita 8, Higashi 4, Higashi-ku, 060-0908 Sapporo, Japan

**Keywords:** Knee osteoarthritis (OA), Center of pressure (COP), Balance, Single‐leg standing, Transition, Patient reported outcome measures, Activities of daily living (ADL), Quality of life (QOL)

## Abstract

**Background:**

Knee osteoarthritis (OA) negatively affects dynamic postural control, which is a basic function that individuals use to perform activities of daily living (ADL). The purpose of this study was to investigate the associations of center of pressure (COP) control during the transition from double-leg to single-leg standing with subjective assessments of ADL and quality of life (QOL) in patients with knee OA.

**Methods:**

Thirty-six patients (29 females) with moderate-to-severe knee OA participated. Dynamic postural control was evaluated during the transition from double-leg to single-leg standing. Each patient stood on a force plate, lifted the less affected limb as fast as possible, and maintained single-leg standing with the more affected limb. The COP movements corresponding to anticipatory postural adjustment (APA) and transitional phases were assessed. The maximum displacement and peak velocity of the COP movements in the medial–lateral direction were calculated. The Knee Injury and Osteoarthritis Outcome Score (KOOS) was used for the subjective assessment of ADL and QOL. Pearson’s product correlation analysis was performed to investigate the associations of COP movements in the APA and transitional phases with KOOS-ADL and KOOS-QOL.

**Results:**

In the APA phase, the maximum COP displacement was significantly correlated with KOOS-ADL (*r* = -0.353, *P* = 0.035) and KOOS-QOL (*r* = -0.379, *P* = 0.023). In the transitional phase, the maximum COP displacement and peak COP velocity were significantly correlated with KOOS-ADL (maximum displacement: *r* = 0.352, *P* = 0.035; peak velocity: *r* = 0.438, *P* = 0.008) and with KOOS-QOL (maximum displacement: *r* = 0.357, *P* = 0.032; peak velocity: *r* = 0.343, *P* = 0.040).

**Conclusions:**

The present study showed that smaller COP movements in the APA phase and smaller and slower COP movements in the transitional phase correlated with poorer ADL and QOL conditions in patients with knee OA. These findings suggest that poor dynamic postural control is associated with poor ADL and QOL conditions in patients with moderate-to-severe medial knee OA. Conservative treatment for patients with knee OA may need to focus on dynamic postural control during the transition from double-leg to single-leg standing.

## Background

Osteoarthritis (OA) of the knee is a common musculoskeletal disorder in elderly individuals [1–3]. Knee OA is one of the main factors that negatively affects individuals’ activities of daily living (ADL) and quality of life (QOL) [4, 5]. Knee OA causes neuromuscular impairments as well as varus knee deformity [6–8]. Neuromuscular impairments can affect ADL and QOL conditions in patients with knee OA and can be improved by rehabilitation [9].

Poor postural control is one of the neuromuscular impairments that occurs in patients with knee OA [6]. Static postural control, which is the ability to stabilize the center of gravity (COG) within a base of support (BOS), is evaluated by measuring the center of pressure (COP) movement, which represents the control of the COG within the BOS [10]. Range and mean velocity of the COP movements during static standing are usually used to evaluate static postural control. Compared with healthy elderly individuals, patients with knee OA exhibit a larger range and mean velocity of the COP movements during static standing [6]. However, some previous studies have reported that static postural control is not associated with ADL or QOL in this patient population [11, 12]. Static postural control may not account for all impairments related to ADL because ADL require COG movements in response to changes in the BOS.

Dynamic postural control, which is the ability to control the COG in response to changes in the BOS, is usually evaluated as the COP displacement and velocity during the transition from double-leg to single-leg standing and gait initiation [13–16]. Recent studies have reported that patients with knee OA exhibit smaller and slower COP movements during gait initiation when compared to healthy elderly individuals [14–16]. These findings indicate that impairments in dynamic postural control are present in patients with knee OA. However, the associations between dynamic postural control impairments and poor ADL and QOL conditions in patients with knee OA remain unknown. The purpose of the present study was to examine the associations of COP movement during the transition from double-leg to single-leg standing with ADL and QOL conditions in patients with knee OA. We hypothesized that the smaller and slower COP movements during the transition from double-leg to single-leg standing would be associated with poorer subjective results regarding ADL and QOL in patients with knee OA.

## Methods

### Study population

Thirty-six patients (29 females and 7 males) with moderate-to-severe medial knee OA planning to undergo total knee arthroplasty participated in this study (Table 1). The sample size was calculated using G*Power 3.1.9.2 (Kiel University, Kiel, Germany). Based on the correlation coefficients between dynamic postural control variables with the Knee Injury and Osteoarthritis Outcome Score (KOOS) for ADL in our first 14 patients (*r* = 0.45; alpha = 0.05; power = 0.80), the total sample size for this study was 36 patients.

The inclusion criteria were as follows: an age of 50 years or older and radiographically diagnosed medial knee OA with Kellgren-Lawrence grade 3–4 in at least one knee [17]. The Kellgren-Lawrence grade indicates the severity of knee OA in terms of osteophyte formation and joint space narrowing using an anterior-posterior knee radiograph during weight-bearing standing, with grade 3 indicating moderate OA and grade 4 indicating severe OA [17]. The exclusion criteria were any previous joint replacement surgery, any back surgery, any neurological disorders that would influence balance, and the inability to safely perform the motion task.This study was approved by the local institutional review board, and each patient provided written informed consent before testing.

**Table 1:** The patients’ demographic and clinical characteristics (n = 36)

Characteristic	Mean (SD)
Age, years	72.2 (8.0)
Height, cm	153.6 (8.5)
Weight, kg	64.3 (11.2)
BMI, kg/m^2^	27.2 (3.9)
Sex, female/male	29/7
KL grade, no. (%)	
Grade 3	15 (41.7)
Grade 4	21 (58.3)
KOOS	
ADL	64.5 (15.1)
QOL	32.7 (17.9)

*SD* standard deviation, *BMI* body mass index, *KL* Kellgren-Lawrence, *KOOS* Knee Injury and Osteoarthritis Outcome Score, *ADL* activities of daily living, *QOL* quality of life

### Transition from double‐leg to single‐leg standing

**Fig. 1:**
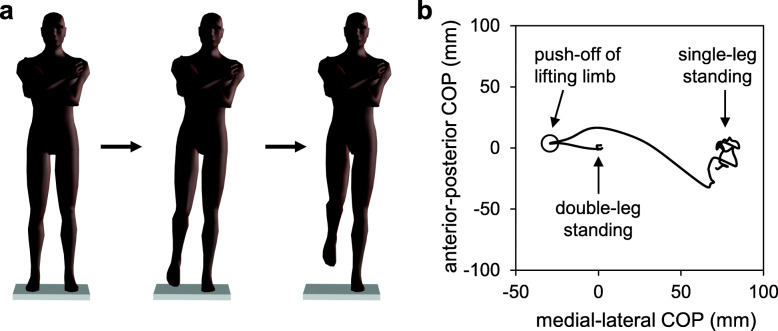
The transition task from double-leg to single-leg standing that was performed to evaluate dynamic postural control (**a**). The center of pressure (COP) first moved toward the lifting limb to push off and then moved toward the stance limb during the task (**b**)

Dynamic postural control was evaluated using the task of transitioning from double-leg to single-leg standing (Fig. 1) [13, 18–21]. The patients were instructed to stand on a force plate (SS-FP40AO-SY; SPORTS SENSING Co., Ltd., Fukuoka, Japan) with the load distributed as evenly as possible between the two legs and to keep looking straight ahead, with their feet shoulder-width apart and their arms folded across the chest. Then, the patients performed the transition from double-leg to single-leg standing as fast as possible after a verbal cue and were asked to maintain single-leg standing as stably as possible for at least 5 s. The force plate data were collected at a sampling frequency of 1000 Hz. The more affected limb was tested in the single-leg standing task. All patients practiced the task at least three times. Data collection started when they became familiar with the task. Each patient was allowed to rest at any time to prevent the effects of fatigue. Trials in which the patients failed to maintain single-leg standing for at least 1 s were excluded from the analysis. Three successful trials performed by each patient were analyzed.

**Fig. 2:**
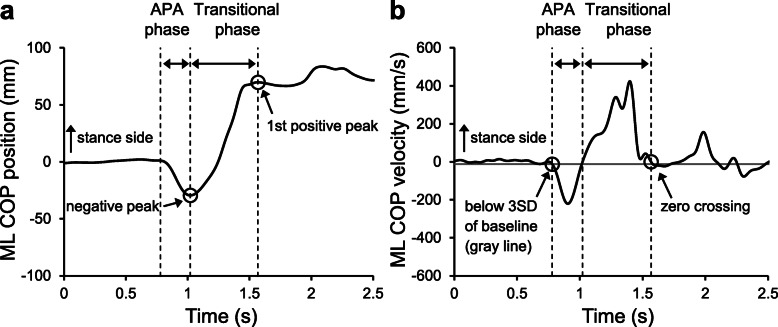
The center of pressure (COP) position (**a**) and velocity (**b**) in the medial–lateral (ML) direction. The positive values indicate the directions toward the stance leg. The circles indicate the onset and end of the anticipatory postural adjustment (APA) and transitional phases

All signals were processed using MATLAB software (version R2014a; MathWorks Inc., Natick, MA, USA). The COP data were filtered using a fourth-order, low-pass Butterworth filter with a cutoff frequency of 10 Hz [13]. This task requires the control of COG movement mainly in the medial–lateral (ML) direction, as the task causes a change in the BOS in the ML direction. Therefore, the COP movement in the ML direction was evaluated. Data analysis was performed for the following two phases (Fig. 2): (1) the anticipatory postural adjustment (APA) phase and (2) the transitional phase. Previous studies have reported that patients with knee OA exhibit impairments in dynamic postural control in the APA and transitional phases [14–16]. In addition, dynamic postural control in the APA and transitional phases is important for single-leg standing [20]. The onset of the APA phase was defined as the first time when the COP velocity exceeded three standard deviations of the baseline for 100 ms. The end of the APA phase was defined as the time of the maximum displacement of the COP toward the lifting leg side [22]. The transitional phase started at the end of the APA phase, and ended with the first peak of the COP signal toward the stance-leg side [22]. The time of the first peak of the COP signal was determined as the time of the zero crossing of the COP velocity. These event times were calculated using a custom MATLAB program. The maximum COP displacement and the peak COP velocity were calculated in the APA and transitional phases. In this study, COP movements toward the stance-leg side were considered positive signals. Therefore, the maximum COP displacement and the peak COP velocity in the APA phase were considered negative, while in the transitional phase, they were considered positive.

### Subjective assessment of ADL and QOL

The Japanese versions of the KOOS subscales were used for the subjective assessment of ADL and QOL [23]. The KOOS subscales have sufficient reliability and validity in Japanese patients with knee OA [23]. The KOOS-ADL subscale consists of 17 questions, and the KOOS-QOL subscale consists of 4 questions. Each patient answered each question on a five-item Likert scale. Higher KOOS subscale scores indicate better conditions. Each subscale ranged from 0 (worst) to 100 (best).

### Statistical analysis

Pearson’s product correlation analysis was performed to investigate the associations of the COP movements during the transition task from double-leg to single-leg standing with KOOS-ADL and KOOS-QOL. The statistical significance level was set at *P* < 0.05. All statistical analyses were performed using IBM SPSS Statistics, version 26.0 (IBM Corporation, Armonk, NY, USA).

## Results

**Fig. 3:**
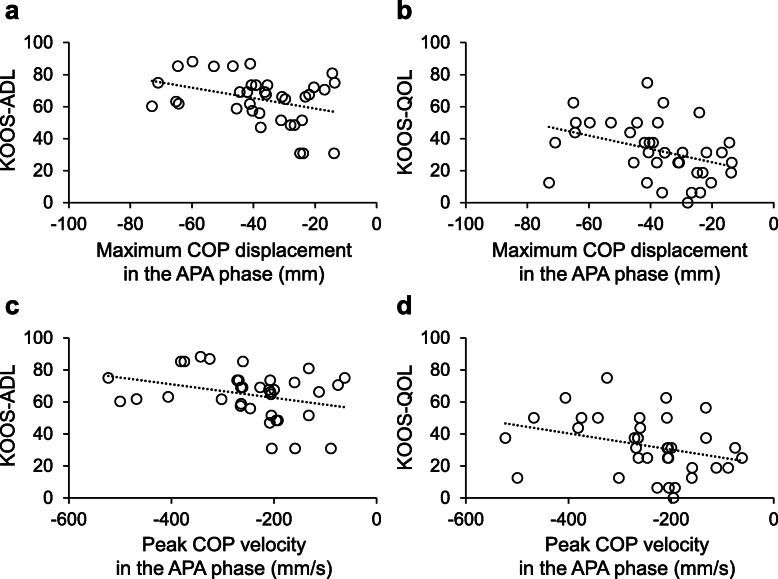
The associations of the maximum center of pressure (COP) displacement and peak COP velocity in the anticipatory postural adjustment (APA) phase with the Knee Injury and Osteoarthritis Outcomes Score (KOOS) subscores for activities of daily living (ADL) and quality of life (QOL)

**Fig. 4:**
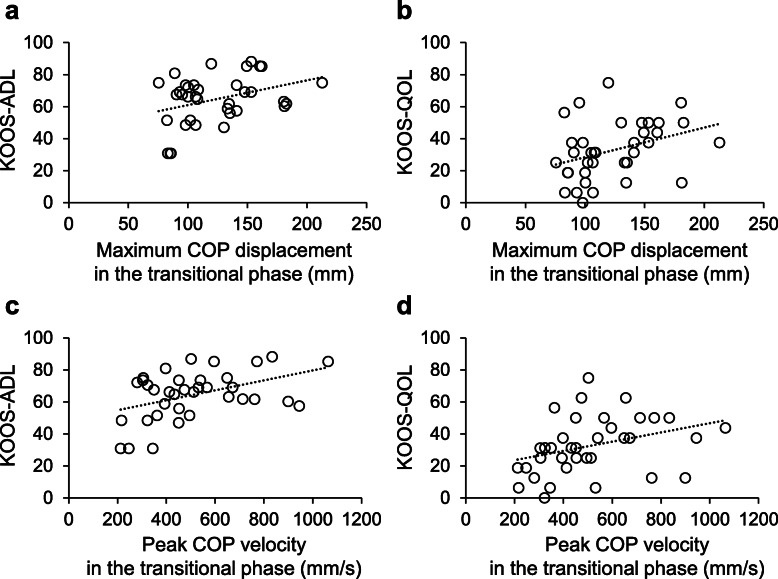
The associations of the maximum center of pressure (COP) displacement and peak COP velocity in the transitional phase with the Knee Injury and Osteoarthritis Outcomes Score (KOOS) subscores for activities of daily living (ADL) and quality of life (QOL)

The maximum COP displacement and peak COP velocity during the transition from double-leg to single-leg standing are shown in Table 2. In the APA phase, the maximum COP displacement was significantly correlated with KOOS-ADL (*r* = -0.353, 95 % confidence interval [CI] = -0.611 to -0.027, *P* = 0.035) and with KOOS-QOL (*r* = -0.379, 95 % CI = -0.629 to -0.057, *P* = 0.023) (Fig. 3a and b). A larger COP displacement toward the lifting leg side in the APA phase was associated with better KOOS-ADL and KOOS-QOL scores. On the other hand, the peak COP velocity in the APA phase was not significantly correlated with KOOS-ADL (*r* = -0.313, 95 % CI = -0.582 to 0.017, *P* = 0.063) or KOOS-QOL (*r* = -0.316, 95 % CI = -0.584 to 0.014, *P* = 0.061) (Fig. 3c and d). In the transitional phase, the maximum COP displacement and peak COP velocity were significantly correlated with KOOS-ADL (maximum displacement: *r* = 0.352, 95 % CI = 0.026 to 0.610, *P* = 0.035; peak velocity: *r* = 0.438, 95 % CI = 0.127 to 0.670, *P* = 0.008) and with KOOS-QOL (maximum displacement: *r* = 0.357, 95 % CI = 0.033 to 0.614, *P* = 0.032; peak velocity: *r* = 0.343, 95 % CI = 0.017 to 0.604, *P* = 0.040) (Fig. 4). A larger and faster COP movement toward the stance-leg side in the transitional phase was associated with better KOOS-ADL and KOOS-QOL scores.

**Table 2:** The COP movements during the transition from double-leg to single-leg standing

Variables^a^	Mean (SD)
APA phase	
maximum COP displacement (mm)	-37.7 (16.3)
peak COP velocity (mm/s)	-247.5 (111.8)
Transitional phase	
maximum COP displacement (mm)	123.1 (34.5)
peak COP velocity (mm/s)	512.2 (212.6)

*SD* standard deviation; *APA* anticipatory postural adjustment; *COP* center of pressure

^a^ Positive values indicate COP movements toward the stance-leg side, and negative values indicate COP movements toward the lifting leg side

## Discussion

The present study investigated the association of COP movements during the transition from double-leg to single-leg standing with KOOS-ADL and KOOS-QOL in patients with medial knee OA. A larger COP movement in the APA phase and larger and faster COP movement in the transition phase were associated with better KOOS-ADL and KOOS-QOL scores. These findings support the *a priori* hypothesis.

The APA phase is the phase in which propulsion forces are generated to move the COG toward the stance-leg side [16, 24]. A small and slow COP movement in the APA phase results in insufficient propulsion for the COG to move toward the stance-leg side. The transition phase is the phase in which the COG actually moves toward the stance-leg side [22]. A small and slow COP movement in the transitional phase results in insufficient COG movements toward the new BOS on the stance-leg side, making it impossible to remain stable during the single-leg standing task. Therefore, larger and faster COP movements in the APA and transitional phases indicate better dynamic postural control [15]. Rogers and Pai reported that the gluteus medius muscle function is important for the transition from double-leg to single-leg standing [25]. In propelling the COG toward the stance-leg side, anticipatory activation of gluteus medius is needed, while in halting the COG on the stance-leg side, greater activation of gluteus medius is needed [25]. Hinman et al. reported that the muscle strength around the hip joint, including the gluteus medius, is weaker in patients with knee OA compared with healthy elderly individuals [26]. Therefore, dynamic postural control during the transition to single-leg standing may have been affected by impaired gluteus medius muscle function in patients with knee OA. In addition, external knee adduction moment, which is believed to be associated with knee pain [27], was observed during the transition from double-leg to single-leg standing [28]. Thus, knee pain may have also affected the small and slow COP movement in the APA and transitional phases. Further research is needed to clarify the factors related to small and slow COP movement during the transition from double-leg to single-leg standing.

In some previous studies, static postural control during double-leg standing was not associated with ADL or QOL in patients with knee OA [11, 12]. Static postural control, which is the ability to maintain balance within a fixed BOS, is rarely required in everyday locomotion [21]. On the other hand, the task of transitioning from double-leg to single-leg standing is the basis of several ADL, such as walking, stair climbing, and dressing, which involve COG movement with a changing BOS [21]. In addition, dynamic postural control during the transition from double-leg to single-leg standing has been shown to be associated with gait mechanics [28]. Therefore, compared with static assessments, dynamic postural control assessments during double-leg to single-leg standing transition may be better for predicting ADL and QOL in patients with knee OA. The single-leg standing duration is often assessed clinically, but a better approach may be to pay attention to the transition to single-leg standing as well. However, the correlation coefficients in the present study were weak to moderate [29]. Further study is needed to consider not only dynamic postural control but also other factors related to ADL or QOL in patients with knee OA.

Previous studies have reported that repetitive movement training improves postural control in healthy individuals [30, 31]. Repetitive movement training improves not only the COP movement but also neuromuscular control in postural control during a simple reaching task [31]. We speculate that repetitive training of the transition from double-leg to single-leg standing may improve neuromuscular control in this patient population, which may lead to improvements in dynamic postural control. The single-leg standing task is often used as a training task for postural control, as it has been shown to improve elderly people’s single-leg standing duration [32]. However, increasing the single-leg standing duration alone may be insufficient for improving self-reported ADL and QOL conditions in patients with knee OA because the single-leg standing duration, which reflects an individual’s ability to maintain balance within a fixed BOS, is used to evaluate static postural control [33]. The present study showed significant correlations between dynamic postural control during the transition to single-leg standing and self-reported ADL and QOL conditions in patients with knee OA although the correlation coefficients were weak to moderate. Therefore, a potentially beneficial approach is to note the transition to single-leg standing in addition to maintaining single-leg standing in conservative treatments for patients with knee OA. In addition, some patients with knee OA are not able to stand on a single leg, and such patients have a higher risk of falls than do healthy elderly individuals [34–36]. It is safe to practice moving the COP in the ML direction, such as the transition from double-leg to single-leg standing, even in patients with knee OA who are not able to remain standing on one leg [37]. The transition from double-leg to single-leg standing may be suitable as an exercise to improve dynamic postural control in patients with knee OA. Additional research is needed to investigate whether training exercises in which the COP is moved in the ML direction, such as the transition from double-leg to single-leg standing, can improve ADL and QOL conditions in patients with knee OA.

Our study has several limitations that should be considered. First, the patients in this study suffered from moderate-to-severe medial knee OA. It is unknown whether the results of this study can be generalized to patients with early-stage knee OA. Second, a sex imbalance was observed among the study population. However, as knee OA is more common in females than in males [1, 2], the results of this study can be generalized to patients with knee OA. Third, we did not monitor the degree of knee pain during the transition from double-leg to single-leg standing. It may have affected the association of the COP movements with ADL and QOL condition in patients with knee OA. Finally, this study was conducted with a cross-sectional design. Additional studies should be conducted to investigate the effects of practicing the transition from double-leg to single-leg standing on ADL and QOL conditions in patients with knee OA.

## Conclusions

Larger COP movements in the APA phase and larger and faster COP movements in the transitional phase during the transition from double-leg to single-leg standing were significantly correlated with better ADL and QOL conditions in patients with knee OA. The present findings suggest that better dynamic postural control is associated with better ADL and QOL conditions in patients with moderate-to-severe medial knee OA. In the assessment and treatment of patients with knee OA using single-leg standing, clinicians may need to focus not only on the maintenance of single-leg standing but also on the transition to single-leg standing.

## Data Availability

The datasets used and/or analyzed during the current study are available from the corresponding author upon reasonable request.

## Abbreviations

OA: Osteoarthritis
ADL: Activities of daily living
QOL: Quality of life
COP: Center of pressure
COG: Center of gravity
BOS: Base of support
ML: Medial–lateral
APA: Anticipatory postural adjustment
KOOS: Knee Injury and Osteoarthritis Outcome Score
